# Neural stem/progenitor cells from adult canine cervical spinal cord have the potential to differentiate into neural lineage cells

**DOI:** 10.1186/s12917-023-03757-3

**Published:** 2023-10-06

**Authors:** Woo Keyoung Kim, Yeon Sung Son, Ji-Hey Lim, Wan Hee Kim, Byung-Jae Kang

**Affiliations:** 1 Department of Veterinary Clinical Sciences, College of Veterinary Medicine and Research Institute for Veterinary Science, Seoul National University, Seoul, 08826 Korea; 2 BK21 FOUR Future Veterinary Medicine Leading Education and Research Center, Seoul National University, Seoul, 08826 Korea; 3 Medical Research Center, College of Medicine, Seoul National University, Seoul, 03080 South Korea; 4 Department of Neurology/Neurosurgery, College of Veterinary Medicine, University of Missouri, Columbia, 65211 USA

**Keywords:** Neural progenitor cells, Dogs, Neurospheres, Differentiation

## Abstract

**Background:**

• Neural stem/progenitor cells (NSPCs) are multipotent self-renewing cells that can be isolated from the brain or spinal cord. As they need to be isolated from neural tissues, it is difficult to study human NSPCs. To facilitate NSPC research, we attempted to isolate NSPCs from dogs, as dogs share the environment and having many similar diseases with humans. We collected and established primary cultures of ependymal and subependymal cells from the central canal of the cervical spinal cord of adult dogs. To isolate pure NSPCs, we employed the monolayer culture and selective medium culture methods. We further tested the ability of the NSPCs to form neurospheres (using the suspension culture method) and evaluated their differentiation potential.

**Results:**

• The cells had the ability to grow as cultures for up to 10 passages; the growth curves of the cells at the 3rd, 6th, and 9th passages showed similar patterns. The NSPCs were able to grow as neurospheres as well as monolayers, and immunostaining at the 3rd, 6th, and 9th passages showed that these cells expressed NSPC markers such as nestin and SOX2 (immunofluorescent staining). Monolayer cultures of NSPCs at the 3rd, 6th, and 9th passages were cultured for approximately 14 days using a differentiation medium and were observed to successfully differentiate into neural lineage and glial cells (astrocytes, neurons, and oligodendrocytes) at all the three passages tested.

**Conclusion:**

• It is feasible to isolate and propagate (up to at least 10 passages) canine cervical spinal cord-derived NSPCs with the capacity to differentiate into neuronal and glial cells. To the best of our knowledge this is the first study to successfully isolate, propagate, and differentiate canine NSPCs derived from cervical spinal cord in the adult canine, and we believe that these cells will contribute to the field of spinal cord regeneration in veterinary and comparative medicine.

**Supplementary Information:**

The online version contains supplementary material available at 10.1186/s12917-023-03757-3.

## Background

Damage to the spinal cord can be devastating, as the neurons in the spinal cord do not regenerate easily, and the resulting loss of sensory and motor function can be permanent [1, 2]. In addition, such spinal cord injuries (SCIs) impose a high societal economic burden [3]. To date, there is no effective treatment for SCI, though this has been a popular topic of research [4, 5].

Stem cell transplantation in SCI has been suggested to have a beneficial effect on reconstructing neural circuits and remyelination [6–8]. Among stem cells, Neural stem/progenitor cells (NSPCs) are a potential source that has been demonstrated in in vivo models, resulting in post-mitotic astrocytes, oligodendrocytes, and neurons [9–12]. Furthermore, NSPCs have the ability to support neural protection, and neurite outgrowth; They also exert anti-inflammatory and antioxidant effects [13, 14]. These points highlight the potential of NPCs, however, it is difficult to study these cells in humans, because human-derived NSPCs must be harvested from neural tissues. Therefore, this study focused on dogs, which share similarities with human SCI with regard to genetic background, severity and location, and pathological processes [15–17].

Ependymal cells lining the central canal of the spinal cord have the ability to differentiate into neural cells in vitro, and are referred to as NSPCs [18, 19]. Although neural stem cells have been isolated from fetal canine spinal cord [20], there is currently no evidence that these cells can be isolated from adult canine spinal cord. In adult human spinal cord, there is a report that ependymal and subependymal cells around the central canal express neural precursor markers [21]. Here, we report on the isolation and culturing of NSPCs from the cervical spinal cord (ependymal and subependymal cells around the central canal) of adult dogs; we also identify the differentiation and self-renewal capacities of these cells for the first time.

## Results

### NSPC morphology

**Fig. 1:**
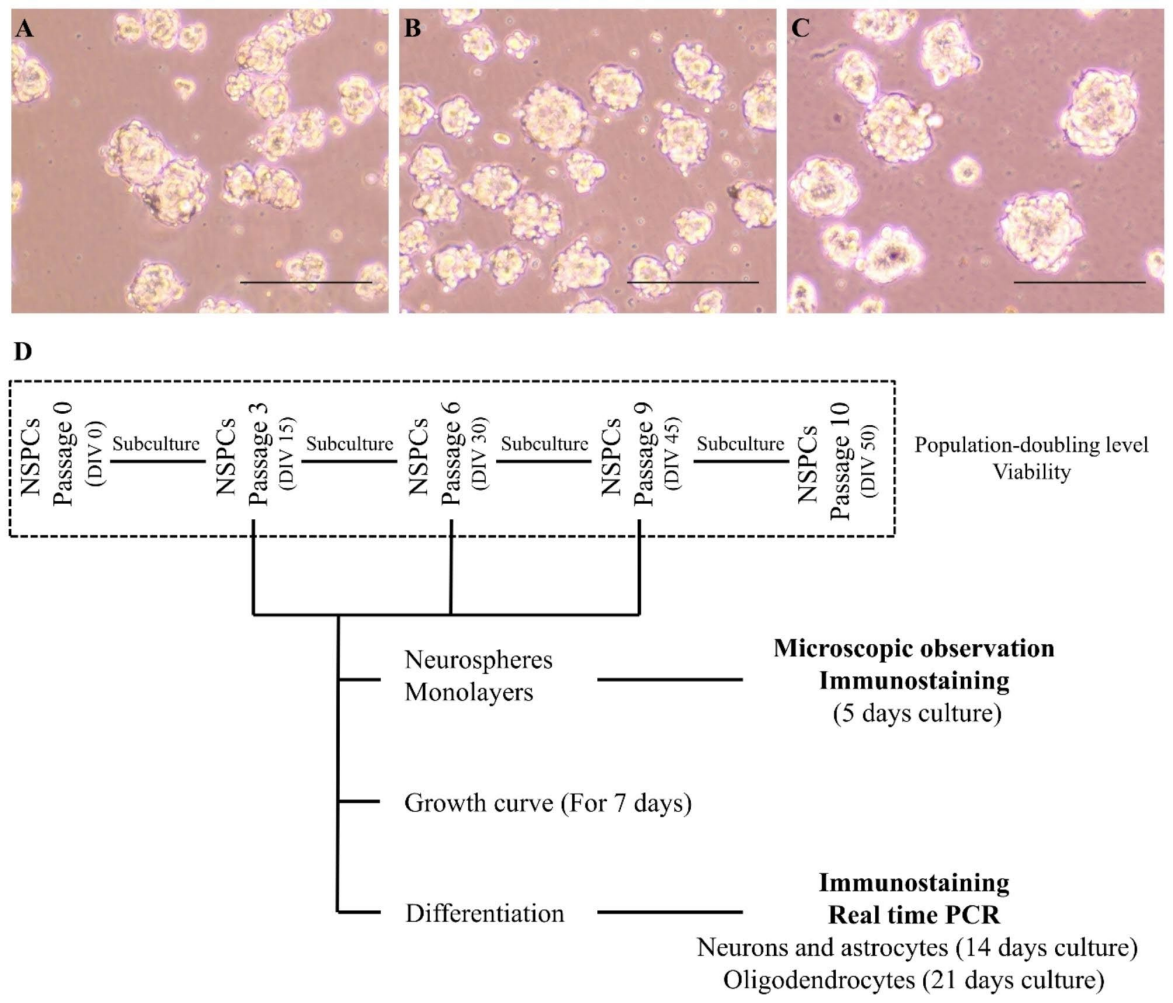
Neurospheres obtained by suspension culture of **(A)** cells at passage 3, **(B)** cells at passage 6, and **(C)** cells at passage 9. The scale bar represents 200 μm. **(D)** Scheme of experiment: neural stem/progenitor cells (NSPCs) were serially passaged in monolayer from passage 0 to 10. Cells that were cultured with monolayer were additionally cultured in neurospheres form only in 3rd, 6th, and 9th passage cells for microscopic and immunostaining observation. Growth curve and cell differentiation analysis were conducted in monolayer

**Fig. 2:**
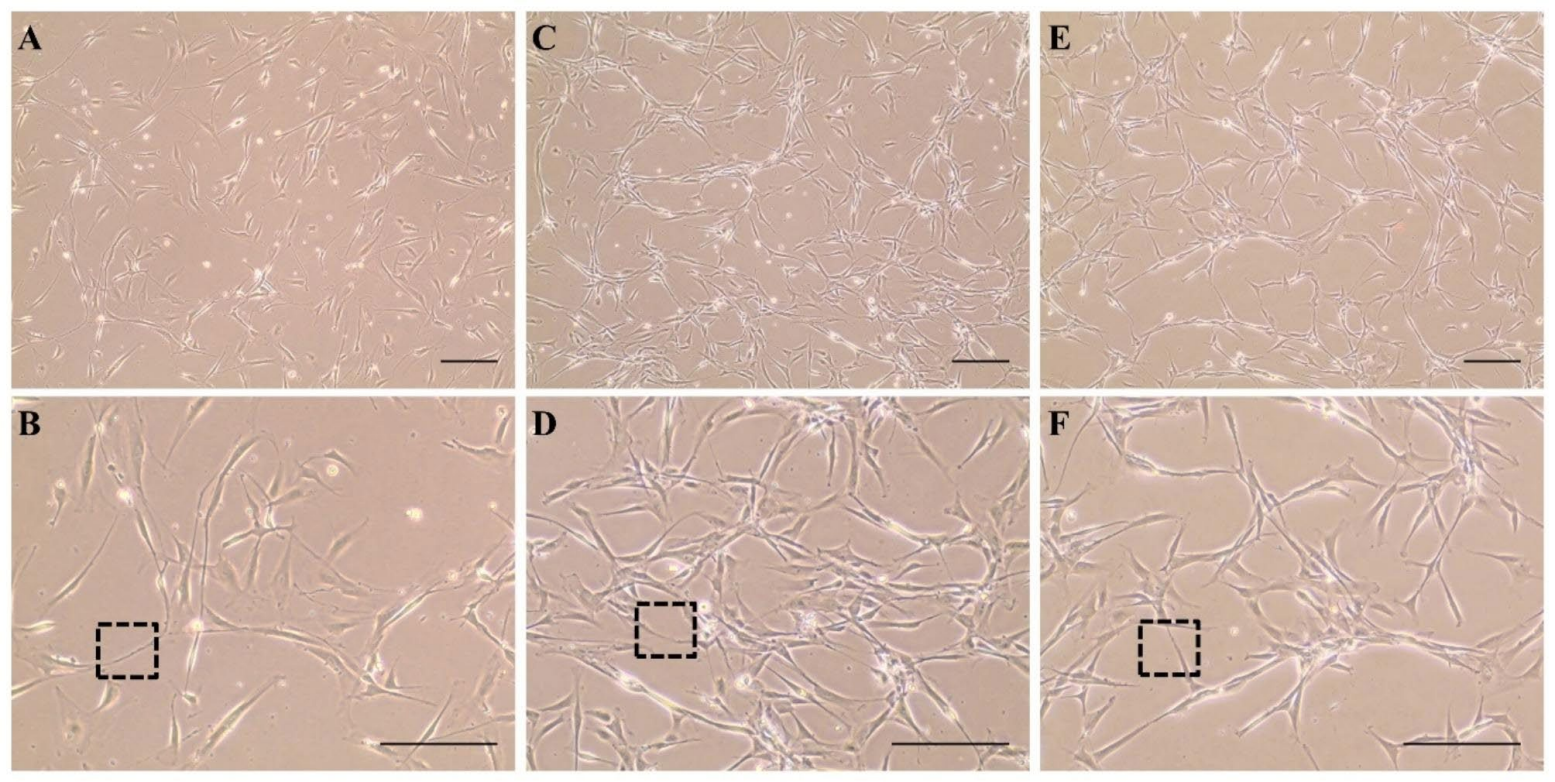
Cervical spinal cord-derived neural stem/progenitor cells (NSPCs) in monolayer culture **(A)** and **(B)** cells at passage 3, **(C)** and **(D)** cells at passage 6, and **(E)** and **(F)** cells at passage 9. NSPCs exhibited intercellular connection by developing cell protrusions. Dotted boxes represent cell protrusions. The scale bar represents 200 μm

Following the collection of the cervical spinal cord from the dogs, a selective isolation of the central canal of the spinal cord was performed, followed by its culture in a suitable medium. The passage 0 cells were initially cultured as neurospheres and subsequently transitioned to a monolayer culture during the subculture. Rounded neurospheres were observed after three days of primary culture of the isolated NSPCs. The diameters of the neurospheres ranged from approximately 50–150 μm (Fig. 1). The neurospheres showed uniformity in size and shape. In monolayer culture, NSPCs adhered to the bottom of cell culture plates and exhibited intercellular connection by developing cell protrusions. Cells at passages 3, 6, and 9 showed similar cell morphology and intercellular connection (Fig. 2).

### Cell growth characteristics and viability

**Fig. 3:**
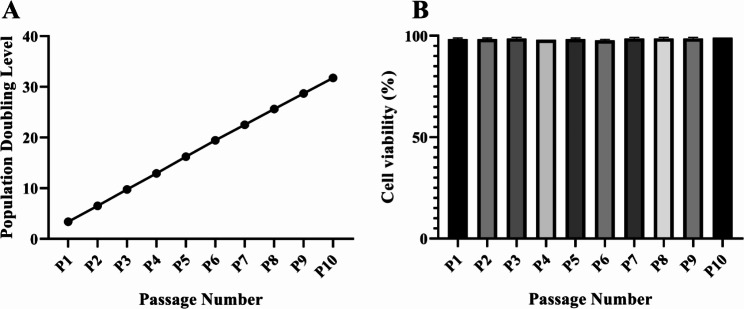
Growth characteristics and viability of neural stem/progenitor cells (NSPCs) **(A)** NSPCs were serially passaged in monolayer for five days until 80–90% confluency at each passage. The population-doubling level (PDL) was measured based on the average of the total number of three plate (60 mm PLO/fibronectin-coated petri dishes) cells in each passage. **(B)** Cell viability was measured based on the average viability of three plate cells in each passage

**Fig. 4:**
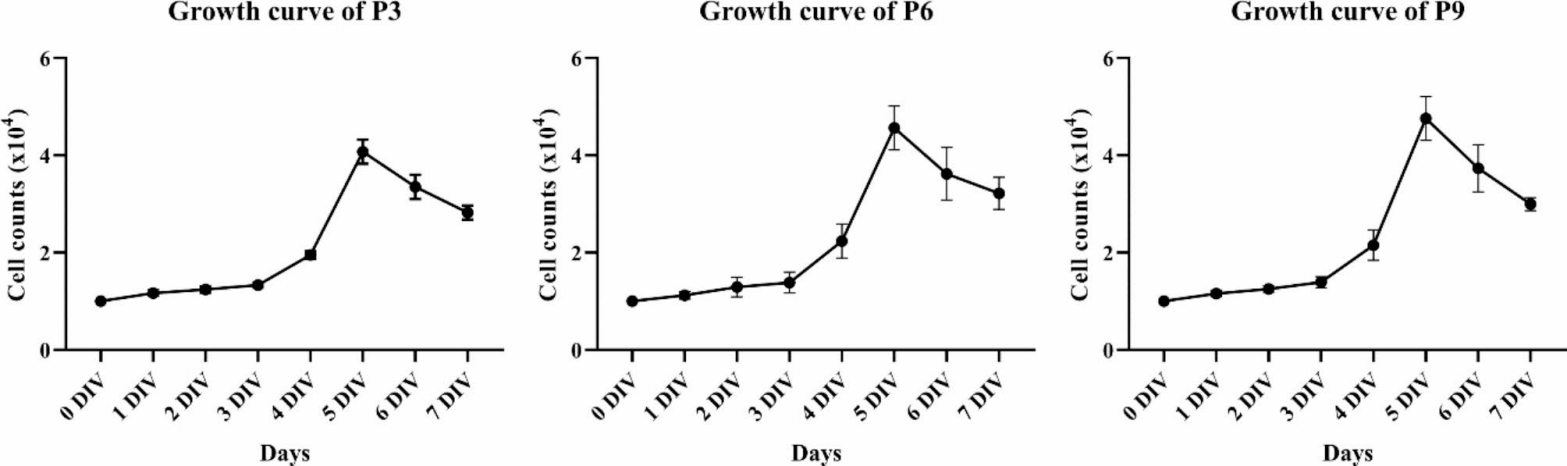
Growth curve of cells at each passage. Each passage of cells (1 × 10^4^) was seeded in each well of 24-well plate. The cell count was measured based on the average of the total number of three well cells. Daily cell counts were performed over seven successive days to construct the growth curve

The NSPCs were serially passaged to analyze their growth characteristics. At each passage, cell counts, and viability assessments were performed using trypan blue staining. The NSPCs maintained their ability to proliferate without any change in the proliferation rate for up to 10 passages (Fig. 3A). Cell viability measurements were performed during passaging and the viability was found to be > 97% at all passages tested (Fig. 3B). The growth curves of the cells at passages 3, 6, 9 are shown in Fig. 4. The NSPCs grew slowly from day 1 to day 3 of culture. From 3 days in vitro (DIV) onwards, the NSPCs showed a logarithmic growth pattern followed by a decrease in the proliferation rate at DIV 5–7 (Fig. 4).

### Characterization of NSPCs by immunofluorescence

**Fig. 5:**
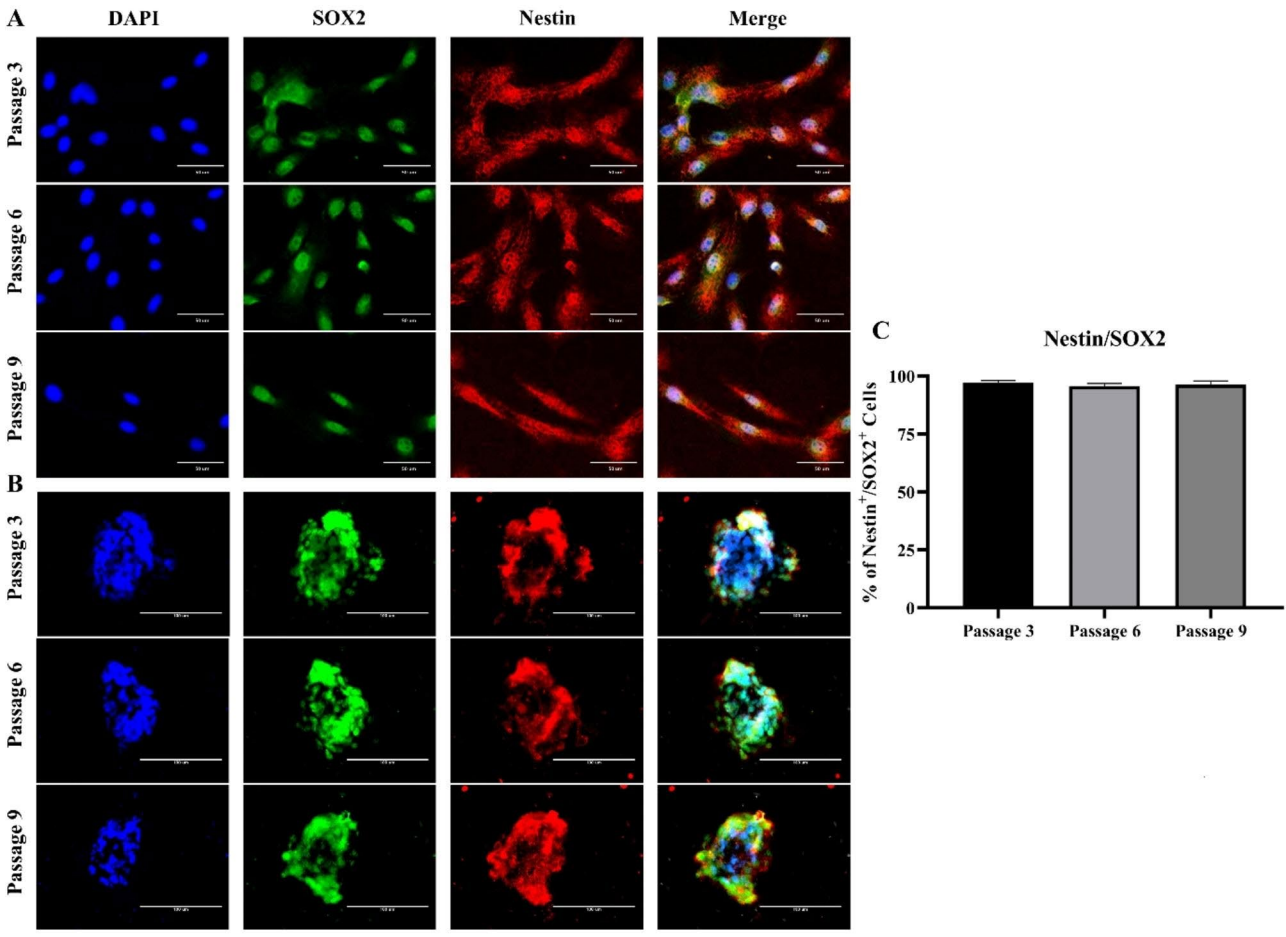
Immunocytochemistry of neural stem/progenitor cells (NSPCs) cultured as neurospheres or monolayers. **(A)** 3rd, 6th, and 9th passage cells cultured in a monolayer for 5 DIV. **(B)** 3rd, 6th, and 9th passage cells cultured as neurosphere for 5 DIV. Nuclei were stained with DAPI (blue) and NSPCs marker for SOX2 (green) and nestin (red). **(C)** Each well of the 24-well plate was immunostained with nestin and SOX2. The quantification was performed by calculating the average percentage of nestin^+^/SOX2^+^ cells out of the total cells in three wells. Quantification was carried out with cells cultured in monolayer

**Fig. 6:**
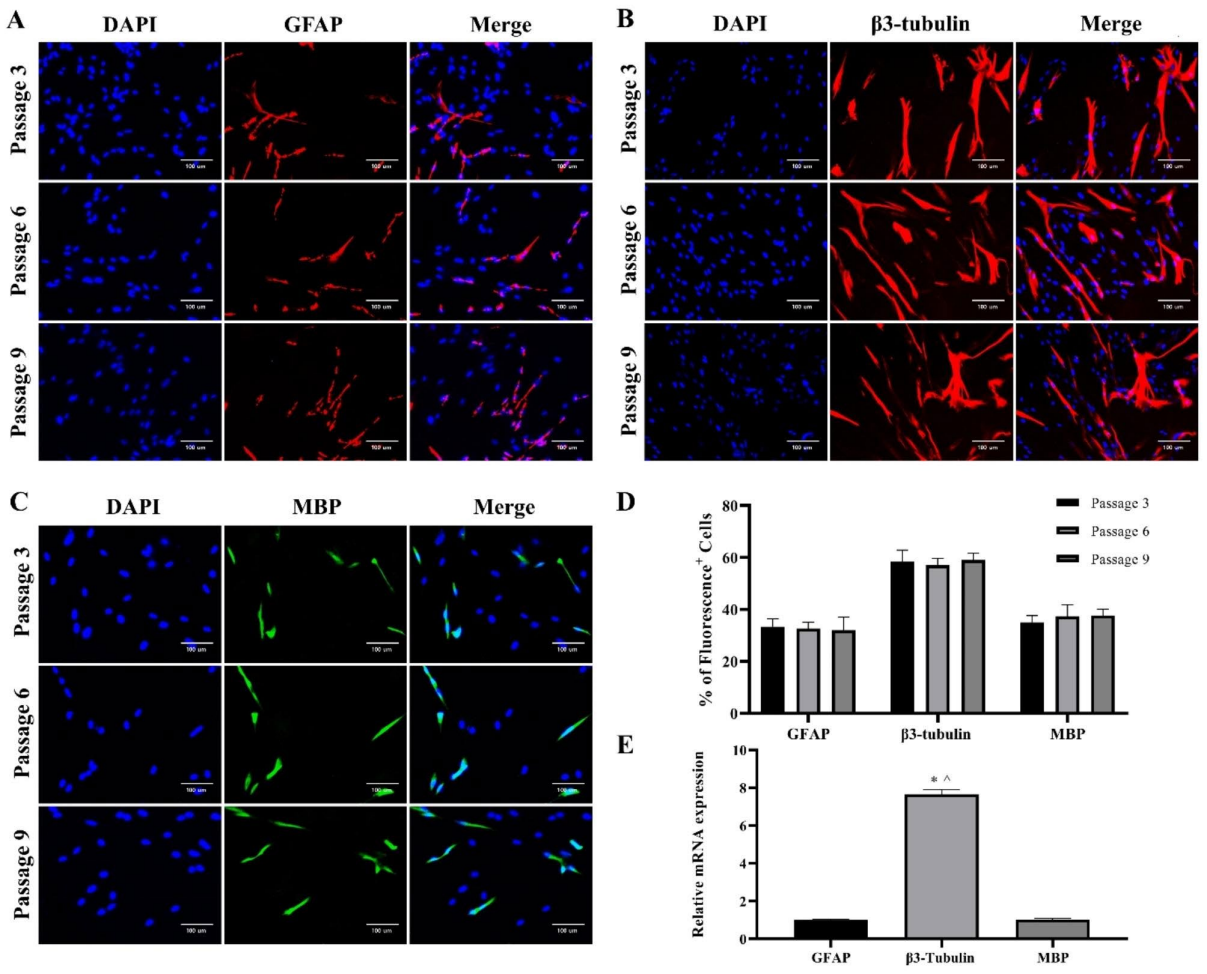
Immunocytochemistry of differentiated neural stem/progenitor cells (NSPCs). Medium 3 was used to differentiate cells 3rd, 6th, and 9th passage cells. **(A)** astrocyte marker glial fibrillary acidic protein (GFAP) (red), culture for 14 DIV. **(B)** immature neuron marker β-3 tubulin (red), culture for 14 DIV. **(C)** mature oligodendrocyte marker myelin basic protein (MBP) (green), culture for 21 DIV. Nuclei were stained with DAPI (blue). **(D)** Each well of the 24-well plate was immunostained with single marker and quantified. The quantification was performed by calculating the average percentage of GFAP^+^, β-3 tubulin^+^, and MBP^+^ cells out of the total cells in three wells. **(E)** Comparison of gene expression of differentiated cells (passage 3). The data were obtained from experiments biologically repeated two times, with each bar representing an average of the gene expression calculated with the formula 2^−ΔΔCT^; CT value was normalized to the housekeeping gene glyceraldehyde-3-phosphate dehydrogenase (GAPDH) and mRNA expression is shown relative to the expression in the GFAP group. The error bar represents standard deviation. * denotes significance compared with the GFAP group at p < 0.05. ^ denotes significance compared with the MBP group at p < 0.05

Immunostaining was conducted on pre- and post-differentiation cells from the 3rd, 6th, and 9th passages. To determine the cell characteristics in the pre-differentiation state, NSPCs cultured as monolayers or spheres were subjected to immunostaining. The cells were positive for the NSPC markers nestin and SOX2 (Fig. 5A, B). In the quantified data, nestin^+^/SOX2^+^ cells were more than 95% in all the three passages (Fig. 5C). To determine the differentiation potential of the cells, the cells were grown in differentiation medium 3. The differentiated cells expressed glial fibrillary acidic protein (GFAP) (an astrocyte marker), β3-tubulin (a neuronal marker), and myelin basic protein (MBP) (an oligodendrocyte marker) (Fig. 6A, B, C). These pre- and post-differentiation markers were expressed in cells at all the three passages. In the quantified data, GFAP^+^, β3-tubulin^+^, and MBP^+^ cells showed approximately 30–40%, 50–60%, and 30–40%, respectively, in all the three passages (Fig. 6D). These results showed a similar pattern in mRNA expression, with β3-tubulin expression being the highest, and GFAP and MBP expression at similar levels (Fig. 6E).

### Real-time PCR analysis of NSPCs

Real-time PCR analysis was carried out to assess the cell differentiation in both early and late-passages cells, as well as to investigate the impact of the differentiation medium on the cell differentiation. The incubation period for mRNA acquisition was undifferentiated cells for 5 days, differentiation into neurons and astrocytes for 14 days, and differentiation into oligodendrocytes for 21 days.

**Fig. 7:**
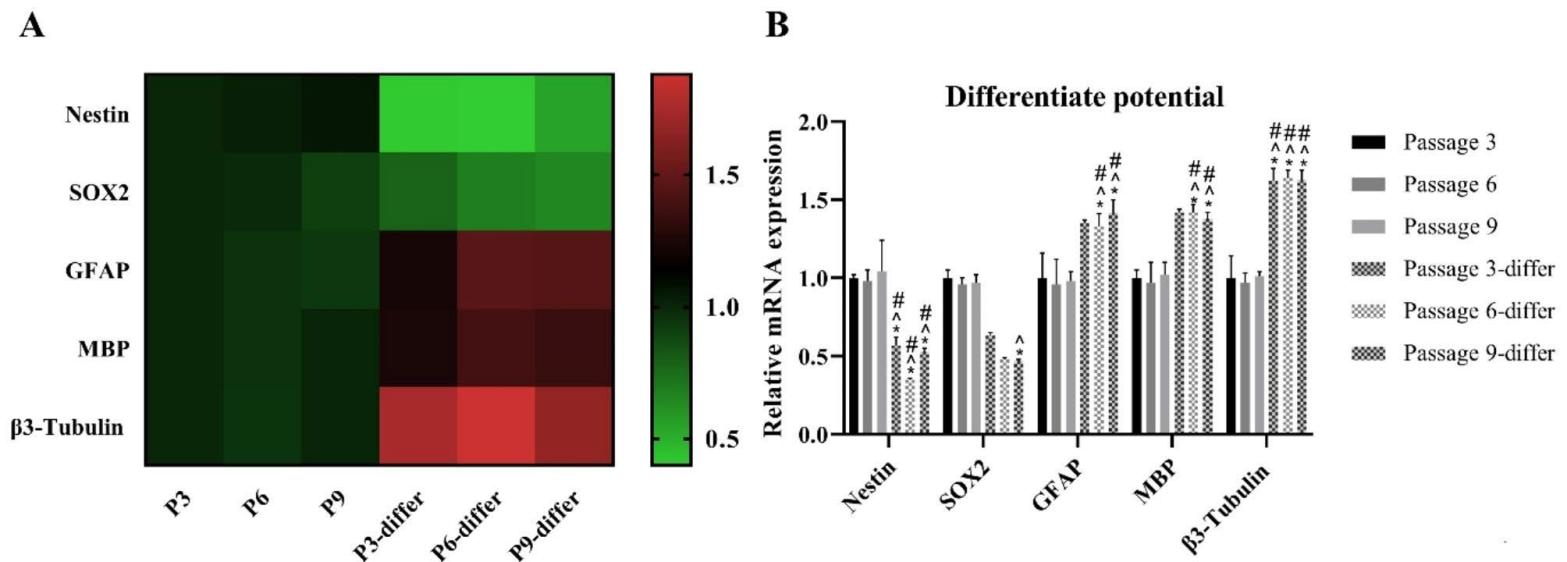
Comparison of gene expression in cells before and after differentiation. Medium 3 was used to differentiate cells. **(A)** Heat map of mRNA expression **(B)** Relative mRNA expression of NSPCs and differentiation markers. The data were obtained from experiments biologically repeated two times, with each bar representing an average of the gene expression calculated with the formula 2^−ΔΔCT^; CT value was normalized to the housekeeping gene glyceraldehyde-3-phosphate dehydrogenase (GAPDH) and mRNA expression is shown relative to the expression in the P3 group (passage 3 cells). The error bar represents standard deviation. * denotes significance compared with the P3 group at p < 0.05. ^ denotes significance compared with the P6 group at p < 0.05. # denotes significance compared with the P9 group at p < 0.05

Real-time PCR was performed to compare gene expression of pre- and post-differentiation cells at early and late passages (Fig. 7). Differentiated cells at passage numbers 3, 6, and 9 exhibited significantly reduced expression of nestin. However, SOX2 expression was significantly decreased only at passage 9. Therefore, the differentiated cells showed reduced expression of pre-differentiation markers. The differentiation-related genes GFAP, MBP, and β3-tubulin were highly expressed in the differentiated cells at all passage numbers. GFAP and MBP expression was significantly high only in cells at passages 6 and 9, but β3-tubulin was significantly high in cells at all the three passages (passage numbers 3, 6, and 9).

**Fig. 8:**
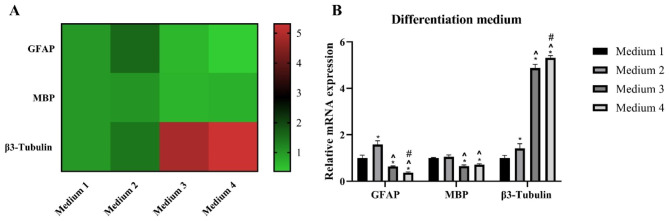
Comparison of gene expression of cells according to differentiation medium. Passage 3 cells was used. **(A)** Heat map of mRNA expression **(B)** Relative mRNA expression of differentiation markers. The data were obtained from experiments biologically repeated two times, with each bar representing an average of the gene expression calculated with the formula 2^−ΔΔCT^; CT value was normalized to the housekeeping gene glyceraldehyde-3-phosphate dehydrogenase (GAPDH) and mRNA expression is shown relative to the expression in the Medium 1 group. The error bar represents standard deviation. * denotes significance compared with the Medium 1 group at p < 0.05. ^ denotes significance compared with the Medium 2 group at p < 0.05. # denotes significance compared with the Medium 3 group at p < 0.05

mRNA is quantified to evaluate gene expression of cell differentiation using FBS and N-2 supplement which is commonly added to the differentiation medium. Differentiation-related gene expression was compared according to the differentiation medium by real-time PCR (Fig. 8). The gene expression of GFAP was the highest in the medium 2 (+ N2) group. In contrast, the medium 3 (+ FBS) and 4 (+ N2 + FBS) groups showed lower GFAP gene expression than the medium 1 (no FBS/N2) and 2 (+ N2) groups. It is considered that N-2 supplement promotes differentiation into astrocytes, but FBS inhibits differentiation into astrocytes. MBP expression was significantly lower in the medium 3 (+ FBS) and 4 (+ N2 + FBS) groups than in the medium 1 (no FBS/N2) and 2 (+ N2) groups. β3-tubulin gene expression was significantly higher in the group cultured in medium 3 (+ FBS) and 4 (+ N2 + FBS), and among them, the medium 4 (+ N2 + FBS) group showed the highest expression. From this, FBS and N-2 supplement are believed to promote differentiation into neurons rather than to oligodendrocytes.

## Discussion

SCI can be irreversible because the CNS has considerably low regenerative capacity [1, 6, 22]. After incomplete SCI, propriospinal circuits can play a role in restoring neuronal function, but regeneration is limited due to the unfavorable environment for cell to survive [23–26]. Therefore, many studies have been conducted on the treatment of SCI by NSPC transplantation [17, 27–29]. However, these cells need to be obtained from CNS tissues, and NSPCs obtained from rodents has limitation of inherent species difference compared to human neural stem cells [30–32].Ultimately, for the treatment of SCI in humans, studies with experimental animals with disease courses similar to those in humans, such as dogs, are needed [15–17]. Accordingly, we performed this study on cervical spinal cord-derived NSPCs from adult dogs; adult dog-derived NSPCs have not yet been studied in the context of stem cell therapy for SCI treatment. Here, we demonstrated the feasibility of isolating cervical spinal cord-derived NSPCs in adult dogs; we successfully cultured these cells and characterized them for the first time.

Various cell culture methods have been reported for culturing NSPCs [33–35]. In this study, ependymal and subependymal cells of central canal were isolated and cells were cultured in petridishes. After cells were first confirmed to form neurospheres, then cultured with a monolayer during successive subcultures. This method provides a uniform growth environment for all cells, including nutrition and oxygenation, to create homogenous cell populations [35–37]. Cells at passage numbers 3, 6, and 9 were examined and showed homogenous cellular morphology; the ability to form neurospheres was also confirmed. The cell viability was more than 97% at all the passage numbers tested. Hence, monolayer culture was considered as an effective way to maintain the properties of NSPCs, as cell debris could be effectively removed through a washing process and uniform nutrition conditions could be provided to cells through an even distribution of the culture medium [36].

We used selective serum-free media with growth factors such as epidermal growth factor (EGF) and basic fibroblast growth factor (bFGF). The serum-free medium inhibits cell differentiation; among the added growth factors, EGF promotes cell survival, while bFGF promotes cell proliferation and exhibits cell protection effects [38–40]. Our results showed that the cells exhibited homogenous morphology and growth patterns at all the three passages examined, and the growth curves were also similar. In addition, both nestin and SOX2 were expressed at similar levels by cells at all the passages examined (passage numbers 3, 6, and 9). It has been reported that when primary cells are cultured in selective medium, cell types other than the target cell type do not survive in the culture environment and gradually disappear [35, 41]. Therefore, the selective medium method was chosen for obtaining pure preparations of canine-derived NSPCs; these cells were confirmed to be multipotent with self-renewal capabilities that could be expanded.

Multipotent NSPCs have the ability to differentiate into both neuronal and glial cells [42, 43]. To confirm whether the NSPCs isolated in this study possessed this capability, cell differentiation was induced using a medium without growth factors, as reported previously [44–46]. We observed the expression of neuronal, astrocyte, and oligodendrocyte markers in NSPCs after the induction of differentiation. In real-time PCR analysis, differentiated cells showed decreased expression of NSPC markers and increased expression of differentiation markers. However, while nestin expression was significantly reduced at all passages, SOX2 expression was significantly reduced only at passage 9. It is believed that cell differentiation is suppressed because the stemness associated with SOX2 is strongly expressed at early passages [47, 48]. In this context, GFAP and MBP, which are differentiation markers, did not show significantly high expression at passage 3. It is possible that this is due to the relatively lower level of differentiation, because SOX2 expression at the early passages was reported to be higher than that at late passages among differentiated cells [49, 50]. These results suggest that the NSPCs were multipotent cells and that the stemness associated with SOX2 was highly expressed in the early passages.

Differentiation is usually induced using a medium without growth factors, though supplements such as N-2 supplement and fetal bovine serum (FBS) are also commonly added [44–46]. The gene expression profiles of differentiated NSPCs in a medium supplemented with N-2 supplement and FBS were investigated to evaluate their ability to differentiate into specific cells. GFAP expression was highest in the cells cultured in medium 2 (+ N2). This is similar to the results reported in a previous study where the cells were differentiated into astrocytes using a medium supplemented with N-2 supplement [51]. FBS is also widely used for the differentiation of NSPCs into neurons [45, 46, 52]. Similar to previous studies, the cells grown in a medium with FBS showed high expression of β3-tubulin; however, the expression of MBP and GFAP was low. Interestingly, the cells cultured in medium supplemented with FBS and N-2 supplement showed the highest expression of β3-tubulin. Considering that N-2 is also used in neuronal differentiation [44, 46], it seems that FBS promotes differentiation into neurons and N-2 has an additional synergistic effect. Our results indicated that using a specific medium composition can promote differentiation of NSPCs into the desired cell type.

In this study, differentiation of NSPCs was confirmed by immunofluorescence staining, and differentiation-related genes according to the differentiation medium were confirmed through real-time PCR. Therefore, differentiation markers for each differentiation medium were not visually confirmed through immunofluorescence staining and only a single marker was used for astrocyte, oligodendrocyte and neural lineage cells in both the immunofluorescence and real-time PCR. In particular, in the case of MBP^+^ cells, extended cellular processes should be seen as mature oligodendrocytes, but considering that they are not, double stain with β3-tubulin is required because they can be seen as myelinating neural type cells. Therefore, in the case of oligodendrocytes, additional characterization is required due to their unusual appearance. In real-time PCR, GAPDH, which is widely used as a reference gene, was used, but additional reference genes were not evaluated. Hence, it is considered that further studies will be needed to compare various fluorescent markers of each neural lineage under various medium conditions and to validate the optimal reference gene panel for real time PCR in canine NSPCs differentiation.

## Conclusion

In this paper we describe the isolation and characterization of NSPCs from the cervical spinal cord of adult canines. We show that adult canine-derived NSPCs can proliferate and differentiate into neural lineage and glial cells. Currently, various cell therapy studies are being conducted for the treatment of SCI, and studying canine spinal cord-derived NSPCs would be relevant in this regard. We believe that the adult canine-derived NSPCs characterized in this study can be used in spinal cord regeneration studies; this is expected to contribute not only to veterinary medicine but also to comparative medicine.

## Methods

### Tissue collection

The spinal cord was collected from euthanized dogs. Two male beagle dogs were used (weights 10.26 and 10.02 kg; age: 1 to 2 years), sourced from experimental animal supplier Saeronbio Inc. (Uiwang, Korea). This study was conducted according to the Animal Care and Use Guidelines (The Institute of Laboratory Animal Resources, Seoul National University). The study on tissue collection was approved by the Animal Care and Use Committee of Seoul National University (SNU-210609-1-1).

### Isolation and culture of NSPCs

For NSPC isolation, the anesthesia protocols for euthanasia were as follows: alfaxalone (2 mg/kg IV; Alfaxan; Jurox), tramadol (4 mg/kg IV, Toranzin; Samsung Pharm. Ind. Co., Seoul, Korea), and acepromazine (10 µg/kg IV, Sedaject; Samu Median Co., Seoul, Korea). The maintenance of anesthesia was done with isoflurane (Ifran; Hana Pharm. Co., Seoul, Korea). Then, dorsal laminectomy was performed at cervical vertebrae 1–5 to expose the spinal cord. After injecting alfaxalone (2 mg/kg IV; Alfaxan; Jurox), 10 mL KCl solution (1 M) was injected to the cephalic vein for euthanasia. After confirming that euthanasia was performed through auscultation, spinal cord collection was carried out immediately, and it took about 1 min. Spinal cord tissue was transferred to 60-mm petridishes and washed with phosphate-buffered saline (PBS) to remove debris and blood vessels. Tissues around the central canal (ependymal and subependymal cells) of the spinal cord were collected under a dissecting microscope. These tissues are homogenized by using a microscissors and manually driven syringe and filtered through a 100-mm-pore nylon mesh. The filtered tissue homogenates were centrifuged at 220 × g for 5 min at 4 °C and then resuspended in culture medium. The culture medium consisted of Dulbecco’s Modified Eagle Medium/Nutrient Mixture F-12 (DMEM/F-12) with GlutaMAX™ (Gibco, Carlsbad, CA, USA), 2% B27 supplement (Gibco), 1% penicillin and streptomycin (PS; Gibco), 20 ng/mL EGF (R&D Systems, Minneapolis, MN, USA), and 20 ng/mL bFGF (R&D Systems).

First, the cells were cultured in a petri dish until the neurosphere was formed. Then, in the subculture, cells were cultured in monolayer. To establish monolayers, petridishes were coated with 15 µg/mL poly-L-ornithine solution (PLO; Sigma-Aldrich, St. Louis, MO, USA) and 1 µg/mL fibronectin solution (Sigma-Aldrich). While growing cells in monolayers, debris was removed through washing with PBS when changing medium. EGF and bFGF in the medium selectively proliferate NSPC while differentiated cells die. Cells were incubated at 37 °C with 20% O_2_ and 5% humidified CO_2_. The medium was replaced every 24 h until 80–90% confluence was attained (approximately five days). For subculture, the cells were disassociated with the StemPro™ Accutase™ Cell Dissociation Reagent (Gibco). This method was carried out up to the 10th passage.

In order to observe neurosphere formation in the 3rd, 6th, and 9th passage, cells from the previous passage that grew in the coating dish were collected and transferred to the uncoated petridishes and cultured. Cells were incubated at 37 °C with 20% O_2_ and 5% humidified CO_2_. The medium was replaced every 48 h by inspecting it under a microscope to ensure that it did not contain a neurosphere.

### Differentiation

PLO/fibronectin-coated 12 mm cover slips were placed in 24-well plates. For differentiation induction, 2 × 10^4^ cells were seeded in each well and allowed to grow. The differentiation into neurons and astrocytes was cultured for 14 days and the differentiation into oligodendrocytes was cultured for 21 days. Four types of differentiation media were used to evaluate the differentiation ability of the NSPCs, as follows: medium 1, DMEM/F-12 with GlutaMAX™ and 2% B27 supplement; medium 2: DMEM/F-12 with GlutaMAX, 2% B27 supplement, and 1% N-2 supplement (Gibco); medium 3: DMEM/F-12 with GlutaMAX™, 2% B27 supplement, and 1% FBS (Gibco); and medium 4: DMEM/F-12 with GlutaMAX, 2% B27 supplement, 1% FBS, and 1% N-2 supplement. In real-time PCR, medium 3, a basic differentiation medium that does not contain other supplements, was used.

### Cell growth and viability

NSPCs were cultured in monolayer for five days until 80–90% confluency was attained, and passaged into three 60 mm PLO/fibronectin-coated petri dishes. At each passage, three plate cells were counted and viability assessments were performed using a Countess FL Automated Cell Counter (Thermo Fisher Scientific, Pittsburg, PA, USA) which is using trypan blue staining. The population doubling level (PDL), a cumulative counting technique, was applied using the following formula: PDL_(n/n−1)_ = log (N_f_/N_0_)/log 2, where n = passage number, N_f_ = final number of cells, and N_0_ = number of cells seeded at passage. To obtain the new PDL(n + 1), PDL(n/n − 1) was added to the previous PDL(n) [53].

For growth curve analysis, cells at the 3rd, 6th, and 9th passage were cultured in 24-well plates (coated 12-mm cover slips were inserted into each well). Cell counts were obtained daily for seven days using trypan blue to derive a weekly growth curve.

### Immunofluorescence

Pre- and post-differentiation 3rd, 6th, and 9th passage cells were immunostained. The cells were fixed using 4% paraformaldehyde in PBS for 10 min at room temperature and then permeabilized with 0.1% Triton X-100 in PBS for 5 min. Blocking was performed using 10% FBS in PBS for 30 min. The following primary antibodies were used: nestin (1:400; sc-33,677; Santa Cruz Biotechnology, Dallas, TX, USA) and SOX2 (1:400; bs-23176R-A488; Bioss, Massachusetts, USA) (neural progenitor markers); GFAP (1:400; sc-33,673; Santa Cruz Biotechnology) (glial marker); MBP (1:400; bs-0380R-A488; Bioss) and β3-tubulin (1:400; sc-80,005; Santa Cruz Biotechnology) (neuronal markers). The cells were incubated with the primary antibodies overnight at 4 °C. After washing 3 times with PBS, Secondary antibodies (1:500; sc-533,656; Santa Cruz Biotechnology) were applied for 1 h at RT. The nuclei were stained with 4,6-diamidino-2-phenylindole (DAPI). The stained cells were then examined under a microscope (EVOS FL Imaging System, Stanwood, WA, USA). To exclude non-specific staining, differentiation markers were confirmed as not expressed in pre-differentiation cells which is cultured for 5 DIV (Supplement 1). Quantification was performed using (Image-J version 1.52a; National Institute of Health, Bethesda, MD, USA) by calculating the average percentage of fluorescence^+^ cells out of the total cells in three out of 24-well plates.

### Real-time PCR

mRNA isolation was performed using QIAzole (Qiagen, Hilden, Germany) according to the manufacturer’s instructions. The purity and concentration of RNA were evaluated using an Epoch reader and Gen 5.2 reader (BioTek, Winooski, Vermont, VT, USA). A Prime Script II First-Strand cDNA Synthesis Kit (TaKaRa, Otsu, Japan) was used to synthesize cDNA. Subsequently, RT-PCR was performed using the ABI StepOnePlus Real-Time PCR system (Applied Biosystems, Foster City, CA, USA). The RT-PCR reaction mixture contained the following: primers (Table 1), cDNA, and SYBR Premix Ex Taq (TaKaRa). Glyceraldehyde-3-phosphate dehydrogenase (GAPDH), a housekeeping gene, was used to normalize the mRNA expression data. Gene expression was compared using the 2^−ΔΔCT^ method [54]. The data were obtained from experiments biologically repeated two times.

**Table 1:** Primer sequences used for real-time quantitative PCR

Target gene	Primer sequence (5′-3′)		Product length (bp)	Accession number
	Forward (Tm)	Reverse (Tm)		
Nestin SOX2 GFAP MBP β3-tubulin	TTCCAACCCTTCTCTCGGCT (59.7) AACCCCAAGATGCACAACTC (58.3) CTGCGGCTAGACCAACTCAC (60.7) GCAGATGTGGAGCAGAACAA (58.4) AGCACGGTATAGACCCCAGT (59.0)	CAAGGGTATCAGGCAAGGGC (60.1) CGGGGCCGGTATTTATAATC (55.8) CCAGATCCAGACGGGCTAAG (59.6) GTCCTCTTGGATGGTCTGGA (58.4) TCCGTGTAGTGACCTTTGGC (58.6)	137 152 187 225 244	XM_025430810.2 XM_038445642.1 XM_038676213.1 XM_038653864.1 XM_038666722.1

### Statistical analysis

Quantitative data was analyzed using GraphPad Prism 8.0.1. and represented by mean ± standard deviation (SD). Two-way ANOVA analyses was performed with Tukey’s multiple comparison. Statistical significance was defined as p < 0.05.

### Electronic supplementary material

Below is the link to the electronic supplementary material.

Supplementary Material 1

## Data Availability

All data generated or analysed during this study are included in this published article.

## Abbreviations

NSPCs: neural progenitor cells
SCI: spinal cord injury
CNS: central nervous system
GFAP: glial fibrillary acidic protein
MBP: myelin basic protein
DIV: days in vitro
EGF: epidermal growth factor
bFGF: basic fibroblast growth factor
FBS: fetal bovine serum
GAPDH: glyceraldehyde-3-phosphate dehydrogenase
PDL: population doubling level
PBS: phosphate-buffered salin
DMEM/F-12: Dulbecco’s Modified Eagle Medium/Nutrient Mixture F-12
PS: penicillin and streptomycin
PLO: poly-L-ornithine solution
DAPI: 4,6-diamidino-2-phenylindole
