# Supplementary material for: Neural stem/progenitor cells from adult canine cervical spinal cord have the potential to differentiate into neural lineage cells
Source: BMC Vet Res. 2023 Oct 6;19:193. doi: 10.1186/s12917-023-03757-3 (PMC10557334; doi:10.1186/s12917-023-03757-3)
Supplement: Supplementary file 1 — Supplementary Material 1 [file 12917_2023_3757_MOESM1_ESM.docx]

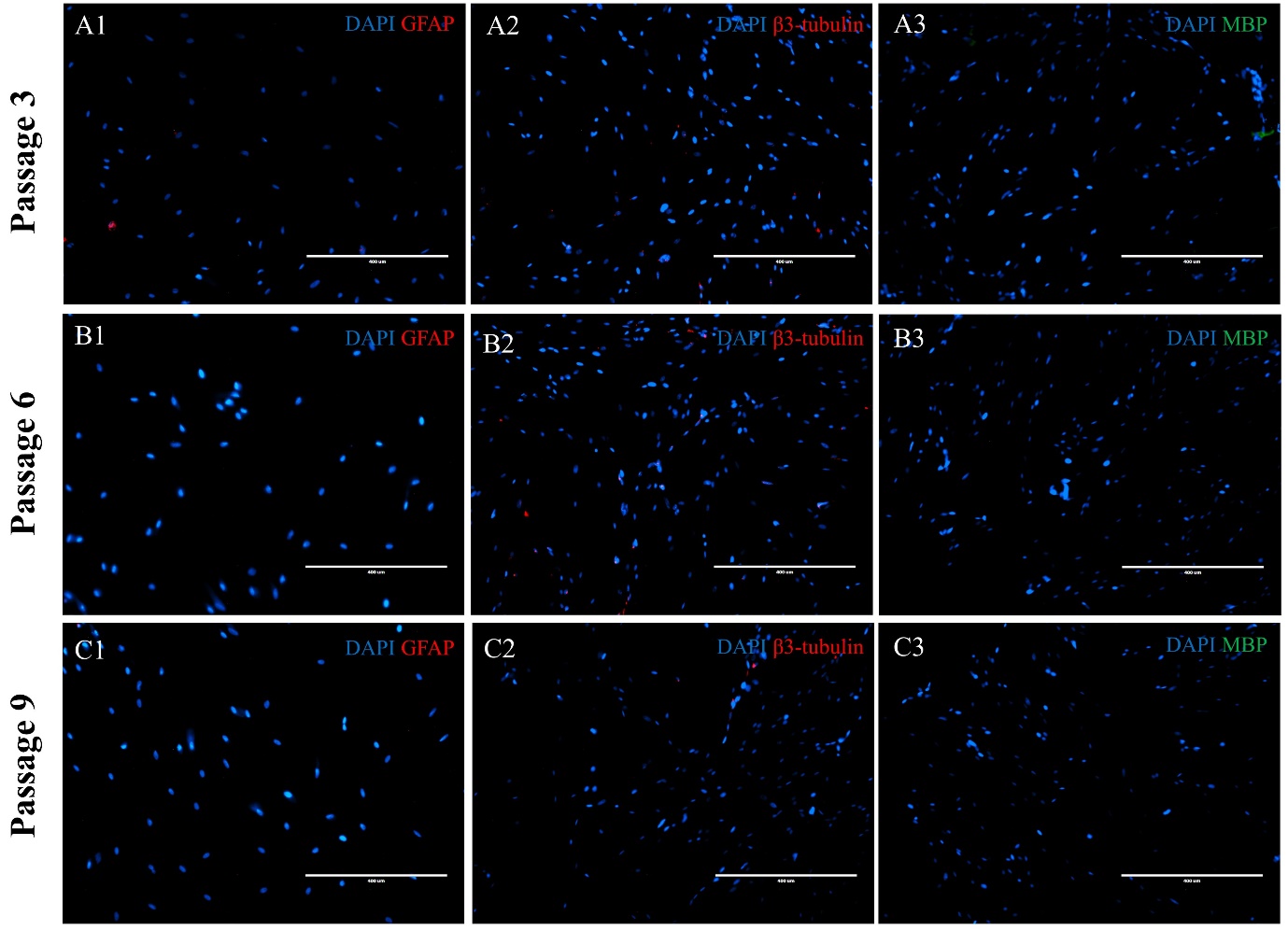


**Supplement 1.** Immunocytochemistry of undifferentiated neural progenitor cells (NPCs) at (A1-A3) passage 3, (B1-B3) passage 6, and (C1-C3) passage 9. Nuclei were stained with DAPI (blue) and the cells were further stained for (A1,B1,C1) astrocyte marker GFAP (red), (A2,B2,C2) immature neuron marker β-3 tubulin (red), and (A3,B3,C3) mature oligodendrocyte marker MBP (green). The scale bar represents 400 μm.
